# PATH - Prediction of Amyloidogenicity by Threading and Machine Learning

**DOI:** 10.1038/s41598-020-64270-3

**Published:** 2020-05-07

**Authors:** Jakub W. Wojciechowski, Małgorzata Kotulska

**Affiliations:** Department of Biomedical Engineering, Wroclaw University of Science and Technology, 50-370 Wrocław, Poland

**Keywords:** Computational biology and bioinformatics, Machine learning, Protein analysis, Protein structure predictions

## Abstract

Amyloids are protein aggregates observed in several diseases, for example in Alzheimer’s and Parkinson’s diseases. An aggregate has a very regular beta structure with a tightly packed core, which spontaneously assumes a steric zipper form. Experimental methods enable studying such peptides, however they are tedious and costly, therefore inappropriate for genomewide studies. Several bioinformatic methods have been proposed to evaluate protein propensity to form an amyloid. However, the knowledge of aggregate structures is usually not taken into account. We propose PATH (Prediction of Amyloidogenicity by THreading) - a novel structure-based method for predicting amyloidogenicity and show that involving available structures of amyloidogenic fragments enhances classification performance. Experimental aggregate structures were used in templatebased modeling to recognize the most stable representative structural class of a query peptide. Several machine learning methods were then applied on the structural models, using their energy terms. Finally, we identified the most important terms in classification of amyloidogenic peptides. The proposed method outperforms most of the currently available methods for predicting amyloidogenicity, with its area under ROC curve equal to 0.876. Furthermore, the method gave insight into significance of selected structural features and the potentially most stable structural class of a peptide fragment if subjected to crystallization.

## Introduction

Amyloids are unbranched, fibrillar protein aggregates, which produce characteristic diffraction pattern in X-ray diffraction experiments^1^. For a long time their occurrence was associated exclusively with severe neurodegenerative diseases, such as Alzheimer’s and Parkinson’s diseases. However, more recent studies showed that these proteins play other roles in a wide variety of organisms, from bacteria and fungi to human^2^. Plenty of studies showed that formation of amyloid fibers depends on the presence of short fragments with an appropriate sequence patterns, called hot-spots^3^. These fragments are responsible for formation of a steric zipper - tightly packed structure which involves two beta sheets that form a core of the amyloid aggregate. High resolution studies, using X-ray diffraction, shed light on molecular details of the steric zipper and revealed different forms of packing peptides into such structures. Theoretical extrapolation, based on symmetry operations, led to proposing ten putative structural classes of the zipper structure, formed by parallel or antiparallel beta sheets^4^. Currently, using X-ray diffraction, seven structural classes of the crystal zipper structure have been identified. Classes 3, 9, and 10 have not been proven, yet.

Amyloidogenic hot-spots can be identified experimentally and computationally. Experiments typically use Congo Red^5^ and Thiflavin T^6^ staining, high resolution techniques such as electron microscopy^7^ and atomic force microscopy^8^. Recently, infrared spectroscopy has become one of the leading methods, due to its simplicity and efficiency^9^. However, experimental techniques are expensive and time consuming, which hampers their use in genome wide studies.

To overcome these limitations several bioinformatic methods for amyloid prediction have been proposed. Some of them, like PASTA 2.0^10^ or ArchCandy^11^, use structural information. Others like Waltz^12^ or AGGRESCAN^13^ employ statistical analysis of a sequence. FoldAmyloid^14^ utilizes density of a protein’s contact sites. Along with a growing number of known amyloidogenic sequences, machine learning methods, such as FISH Amyloid^15^, APPNN^16^, or AmyloGram^17^ were proposed. Finally, consensus predictors, such as MetAmyl^18^ or Amylpred2^19^, are also available. Machine learning methods benefit from statistically significant patterns, which can be found in datasets, capable of providing predictions with a good accuracy. Not all of them reveal relations between the features representing solutions to the problem. For example, most of the bioinformatic methods do not provide much insight into structure of amyloidogenic fragments, especially when aggregates are formed by short sequences. Studying short peptides is relevant, since they represent the phenomenon of amyloidogenicity triggering amyloid pathways of longer peptides or proteins. Moreover, most of the currently known amyloidogenic sequences are hexapeptides^20,21^. Despite polymorphism in amyloid structures, amyloid crystals represent the ground state of the protein folding energy landscape in short peptides, hence including them in modeling amyloidogenicity may bring essential knowledge into these methods^22^.

Accordingly, we combined a structural approach to modeling amyloidogenicity with machine learning methods, and developed PATH (Prediction of Amyloidogenicity by THreading). While classifying amyloidogenic propensity of peptides, PATH should provide structural insight into steric zipper structures formed by their crystals. Furthermore, we aimed to identify the most important energy terms characterizing these structures, which split them between potential amyloids and non-amyloids.

## Methods

### Data set

The data that we used in our study included four data sets of hexapeptides. Peptide fragments of this length are regarded as very good representatives of amyloid hot-spots, which are believed to include between 4 and 10 amino acids. Moreover, they constitute the majority of instances in databases of amyloidogenic sequences.

The first data set, *Templates*, consisted of structural templates that were applied to modeling potential structures of amyloid aggregates formed by other hexapeptides. Based on the structural classification of amyloid hexapeptides, proposed in ^4^, seven crystallographic structures of steric zippers were selected from the Protein Data Bank. Their crystallographic structures of steric zippers were selected from the Protein Data Bank. Each of them represented one of the experimentally confirmed structural class of amyloid hexapeptides (see Table 1). For the purpose of this study, the available structures were processed. All non-protein fragments, such as small organic molecules assisting crystallization, ions, or water molecules were removed from the structures. Since the original structures differed in numbers of chains forming the zipper, our final templates were built with six peptide chains in each beta sheet. In this procedure, copying existing chains and translating them by an appropriate vector was performed, based on the crystallographic data of the original structures.

**Table 1:** Hexapeptide structures representing different classes, used as templates for modeling (class numbering in accordance with^4^).

Structural class	PDB code	Sequence	Origin
1	1YJO	NNQQNY	yeast prion Sup35
2	2Y3J	AIIGLM	amyloid-beta
4	2ONV	GGVVIA	amyloid-beta
5	3LOZ	LSFSKD	beta 2 microglobulin
6	3PZZ	GAIIGL	amyloid-beta
7	3OW9	KLVFFA	amyloid-beta
8	3NHC	GYMLGS	human prion PrP

In the first stage of our study, we modeled structural classes of amyloidogenic peptides. For this purpose we collected a set of validating structures from the Protein Data Bank. The obtained set, *Amyloid Structures*, consisted of 24 amyloid fibers with experimentally determined structures, which were available in the Protein Data Bank and already assigned to a structural class^4^.

The data used in the final classification between two classes (amyloids and non-amyloids) constituted the *Classification set*, which was extracted from the Waltz database^23^. It consisted of 1080 unique hexapeptides, experimentally assigned either to amyloidogenic (244 peptides) or non-amyloidogenic (836 peptides) class. These data were used for design, training, and testing the final effectiveness of our method. Before training the method, the classification set was divided into two separate sets - the training and testing data. During development of the classification methods, k-folds cross validation was performed with k = 5, on the training data set only. The final testing set consisted of 326 randomly selected peptides (30% of the total classification set), in which 85 were amyloidogenic and 241 non-amyloidogenic. It was used to evaluate the complete method.

Our approach was additionally tested with with the use of hexapeptides included in another benchmark set, *pep424*, applied by the authors of Pasta 2.0 for evaluation of their method^10^. The benchmark set consisted of 164 hexapeptides from pep424, in which 67 were amyloidogenic and 97 non-amyloidogenic. The advantage of this data is such that it is much better balanced with regard to representation of both classes. Similarly to our previously described classification test, it was used to train and then test the potential of our algorithm. In both cases the sets were randomly divided into training and test sets containing 70% and 30% of samples, respectively.

### Modeling

Each query sequence, from the set of amyloid structures and from training part of the classification set, was threaded into seven previously described templates. The structural modeling was performed with Modeller 9.21, which is designed for homology or comparative modeling, and its automodel class with default parameters and the model-multichain.py procedure^24^. Ten models were obtained for each structural class (70 models in total), for each of the query sequences. All of the models were scored using DOPE statistical potential implemented in Modeller. The model with the lowest value of this score, representing each of the sequences, was then chosen for further analysis.

First, based on the best model structures obtained from Modeller, we attempted to predict structural classes of peptide fragments. In this study, only 24 peptides included in the set of amyloid structures were modeled.

**Figure 1:**
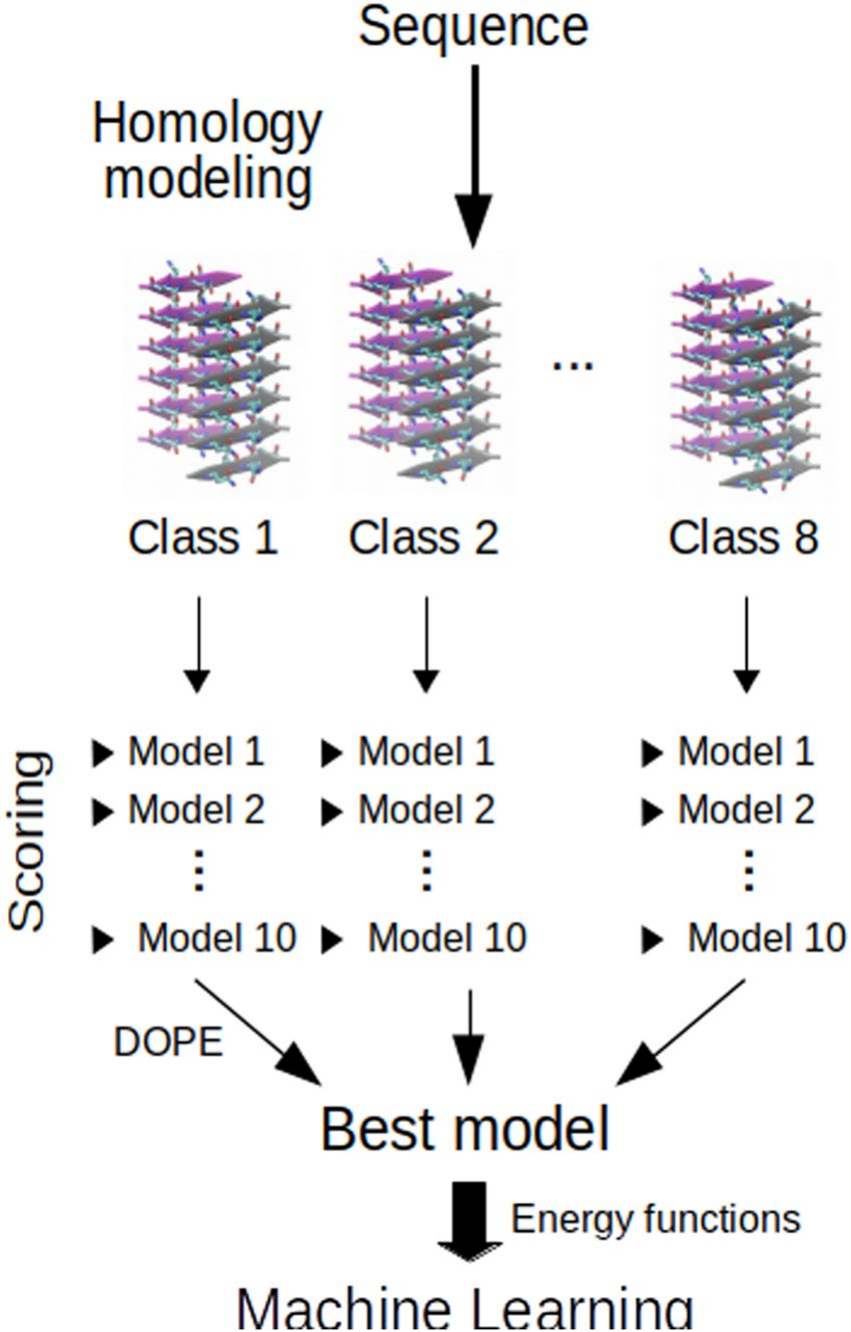
Prediction procedure. Using comparative modeling, query sequence was threaded into seven templates representing different structural classes (class numbering in accordance with^4^). For each of them ten models were proposed and the model with the lowest DOPE score was selected. For this model REF15 and selected PyRosetta energy terms were calculated and used as an input for machine learning classifiers.

The next study was applied to the sequences from the training part of the classification set. For these sequences, Rosetta Energy Function (REF15)^25^ and some of its components were calculated for each of their optimal model structures selected in the first stage. The statistical potentials corresponded to the following energy terms: van der Waals interactions (fa_atr, fa_rep, fa_intra_rep), electrostatic interactions (fa_elec), interactions with a solvent (fa_sol, lk_ball_wtd, fa_intra_sol_xover4), and statistical parameters describing amino acid conformation (omega, fa_dun, p_aa_pp, ref, rama_prepro). All of them were calculated using PyRosetta^26^. These terms, as well as previously computed DOPE and other scores provided by Modeller, were normalized and further used as an input for machine learning classifiers (see Fig. 1). Logistic regression, support vector machines (SVM) with three different kernel functions, and random forest methods were tested.

All classifiers were built using scikit-learn Python library^27^. The logistic regression model with L1 regularization was built using *LogisticRegression* function with default parameters. SVM with linear, polynomial and RBF kernels were built using sklearn.svm.SVC method with default parameters. The random forest consisted of 100 decision trees with the maximum depth of 4, and it was built with *RandomForestClassifier* using cross-entropy as the loss function. Other parameters of models were default. To make sure that the methods do not overfit to the data, k-folds cross validation was performed on the training data set, with k = 5.

Methods were trained and tested on both classification and benchmark sets. In both cases the sets were randomly divided into training and test sets containing 70% and 30% of samples, respectively. To assess the performance of the method, accuracy, which is defined as a fraction of correctly classified samples, sensitivity, specificity, Area Under ROC Curve (AUC) and Matthew Correlation Coefficient (MCC) were calculated.

### Feature selection

In order to identify the most important features that distinguish models of amyloids from non-amyloids, feature selection was performed, using three approaches. In the first step, we analyzed coefficients of logistic regression. In general, input parameters with the largest absolute values of coefficients contribute more to the classification than parameters with coefficients close to zero. Furthermore, the proposed model used L1 regularization, which penalized the contribution of less significant inputs. Feature selection was performed using previously described random forest classifier and feature_importances_ method. Finally feature selection was performed using the algorithm Boruta^28^.

## Results and Discusion

**Figure 2:**
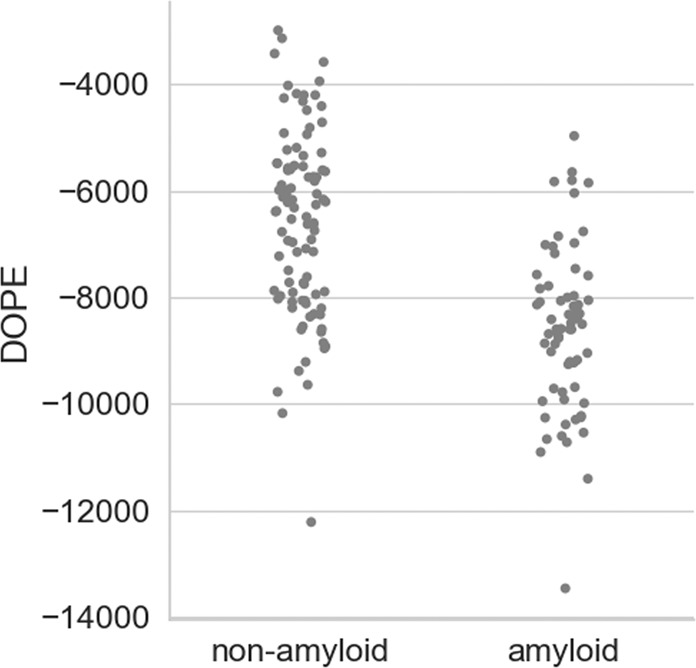
DOPE score of amyloidogenic and non-amyloidogenic sequences threaded onto steric zipper structures for the benchmark set.

Hexapeptides were modeled using seven templates representing all different structural classes of the steric zipper form. For each sequence the model with the lowest DOPE score was chosen. Figure 2 shows the obtained DOPE values for the best models of amyloidogenic and non-amyloidogenic fragments from the benchmark set. As expected, amyloids obtained lower scores when threaded onto steric zippers, which indicate that they formed more energetically favourable, and thus more stable, structures. However, this method alone does not allow unambiguously distinguishing between amyloids and non-amyloids since both classes partially overlap (Fig. 2). Nevertheless, this initial study showed that DOPE may give some clue to the nature of a query hexapeptide.

### Structural class prediction

Based on the comparative modeling and available class templates, we tested whether it is possible to predict the potentially most probably structural class which a query peptide sequence could assume. This hypothesis assumed that the model structure with the lowest energy, represented by DOPE value, should be the most stable and closest to the native structure. Our results showed that the model with the lowest DOPE score did not always correspond to the experimentally observed class. A predicted structural class matched the one experimentally derived in only 11 out of 24 peptides from the set of structures. Table 2 shows the accuracy of the classification in the form one vs all.

**Table 2:** Accuracy of class prediction for different classes from the study on the set of Structures.

class	1	2	4	5	6	7	8
number of peptides	5	4	3	2	1	4	5
accuracy	0.40	0.75	0.67	0.50	0.00	0.25	0.40

Although the results turned out below the expectations, it should be noted that the testing set was very small. Unfortunately, there are very few X-ray structures of amyloid hexapeptides that have been annotated to representative classes^4^, therefore many structural classes were strongly underrepresented. There are also other reasons why such methods might struggle to identify the correct class. For example, most statistical potentials including DOPE are parametrized using a set of globular proteins. Thus, they might describe protein aggregates, such as amyloids, with a certain level of inaccuracy. Another reason may be structural polymorphism of amyloid structures. To certain extent, the final structure of a fiber depends on the experimental conditions, and even in the same environment a population containing different structures can be observed^29^. However, there are studies showing that crystal structures of short amyloid fragments assume a very stable and well defined structure, in contrast to polymorphic fibrils^22^. In the case of our studies, energy differences between amyloids and non-amyloids were much greater than between different structural classes. Therefore, a certain inaccuracy in the class prediction should not affect prediction of amyloidogenicity. Energy differences between amyloids and non-amyloids were much greater than between different structural classes.

### Predicting amyloidogenicity

**Figure 3:**
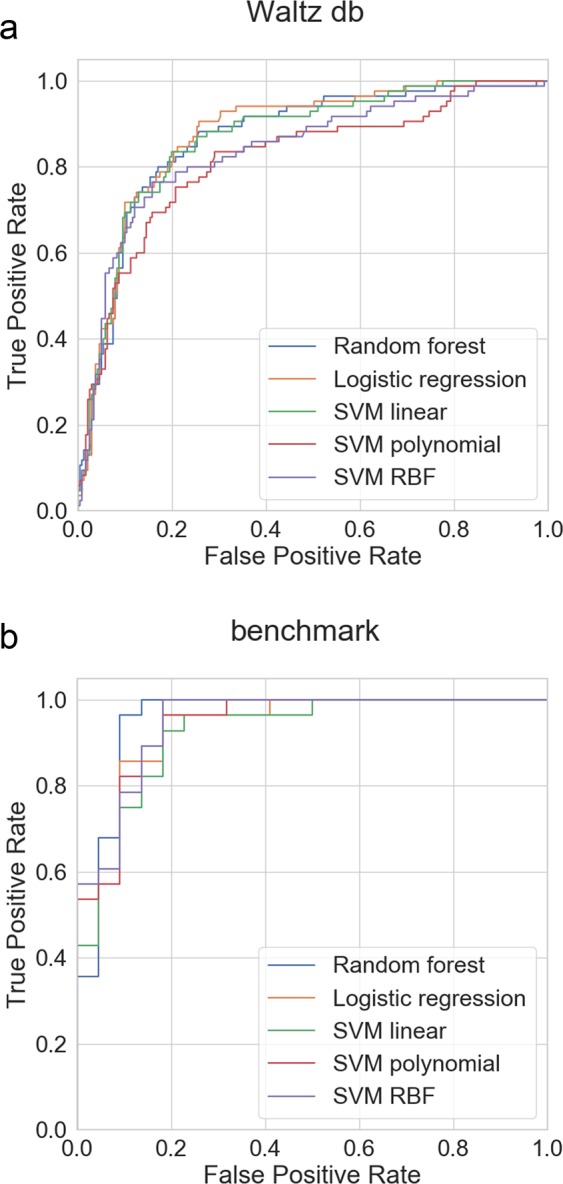
ROC curves for compared machine learning methods, for two test sets.

Using calculated structural scores and several energy terms of the assumably most stable structural models, which were obtained in the first stage, we trained several machine learning methods to classify amyloidogenicity of hexapeptides. Figure 3 shows the receiver operating curves (ROC) of all methods. Table 3 shows metrics of the best classifiers, such as area under ROC curve (AUC), sensitivity and specificity, obtained on the testing subsets of the data used to build our method. These results are very close to the results obtained from the k-fold cross-validation. The performance of selected methods was within the same range. Finally, for PATH we chose logistic regression, which was the simplest and one of the best performing classifiers. An additional benefit of using this method is that it is highly interpretable because it fits several coefficients of the linear function during the training. All methods were trained and tested on the training subsets of two data sets: classification database and hexapeptides from pep424 data set (see Methods).

**Table 3:** Performance of machine learning classifiers trained and tested on subsets of classification (Waltz database) and benchmark sets.

Method	AUC [95% CI]		Sensitivity [95% CI]		Specificity [95% CI]	
	Waltz db	benchmark	Waltz db	benchmark	Waltz db	benchmark
Logistic regression	0.8762 [0.8351–0.9161]	0.9379 [0.9091–0.9609]	0.4235 [0.3082–0.5404]	0.8569 [0.7945–0.9077]	0.9414 [0.9095–0.9712]	0.9069 [0.8528–0.9528]
SVM linear kernel	0.8681 [0.8208–0.9115]	0.9232 [0.8899–0.9500]	0.3540 [0.2597–0.4578]	0.7828 [0.7109–0.8403]	0.9551 [0.9271–0.9811]	0.8655 [0.8024–0.9170]
SVM polynomial kernel	0.8165 [0.7577–0.8732]	0.9418 [0.9153–0.9645]	0.2132 [0.1219–0.3084]	0.6792 [0.6095–0.7449]	0.9792 [0.9577–0.9955]	0.9089 [0.8565–0.9540]
SVM RBF kernel	0.8375 [0.7828–0.8867]	0.9479 [0.9237–0.9670]	0.4843 [0.3681–0.5951]	0.7865 [0.7222–0.8418]	0.9409 [0.9087–0.9710]	0.8627 [0.8000–0.9134]
Random forest	0.8668 [0.8193–0.9090]	0.9544 [0.9289–0.9780]	0.4273 [0.3188–0.5409]	0.8915 [0.8403–0.9387]	0.9259 [0.8929–0.9585]	0.9086 [0.8542–0.9526]

95% confidence intervals (CI) were calculated using bootstrap.

The performance of PATH (on its test set) was compared to selected three other top predictors of amyloidogenicity (Table 4). The performance metrics of other methods are based on the data reported by their authors, calculated using their test sets. The observed differences between the three best performing predictors were negligible. PATH performed very well compared to the methods used here for a comparison.

**Table 4:** Comparison of PATH with several state of the art predictors of amyloidogenicity.

Method	AUC	Sensitivity	Specificity	MCC
PATH	0.8762	0.4235	0.9414	0.4444
Pasta 2.0	0.8550	0.3826	0.9519	0.4291
AmyloGram	0.8856	0.6779	0.9037	0.6057
FoldAmyloid	0.7531	0.7517	0.7185	0.4526

PATH showed one of the best performances among tested methods.

Additionally, as suggested by one of the reviewers, we tested the approach from PATH on FVFLM pentapeptide, which was shown to inhibit aggregation of Abeta amyloid involved in the Alzheimer’s disease, although it is strongly amyloidogenic itself^30^. This peptide was too short for many predictors of amyloidogenicity. Moreover only four out of more than ten predictors that could analyze it, identified it correctly as amyloidogenic. PATH was able to classify it correctly as an amyloid, even though our method was not trained on any pentapeptides or hexapeptides that included this sequence.

### Feature selection

**Figure 4:**
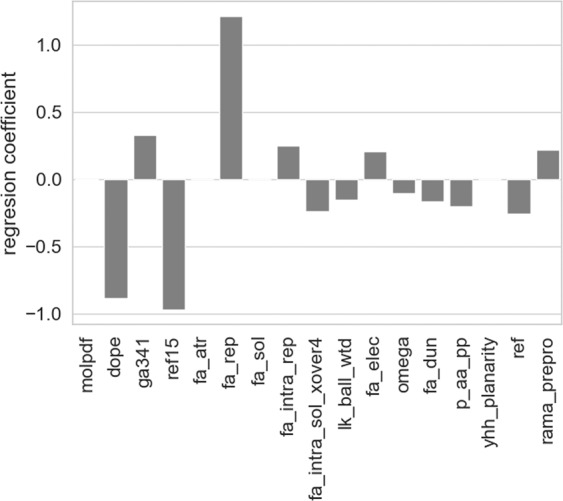
Coefficients of logistic regression model for energy terms.

In the next step, we identified the most important features for our model. This was done by studying the logistic regression coefficients (see Fig. 4). Three most important features turned out to be DOPE score, REF15 score and the repulsive component of Lennard-Jones term (fa_rep). The first two terms represent complex energy functions that are not trivial to interpret. In general, both of them should approximate the free energy of a protein, thus low values of these functions indicate highly stable conformations. As expected, amyloidogenic fragments obtained lower scores when threaded into a steric zipper structure. The last identified term, fa_rep, has a physical interpretation - it describes repulsion between atoms, arising from Pauli repulsion. This indicates that structures with high values of this function are too tightly packed.

**Figure 5:**
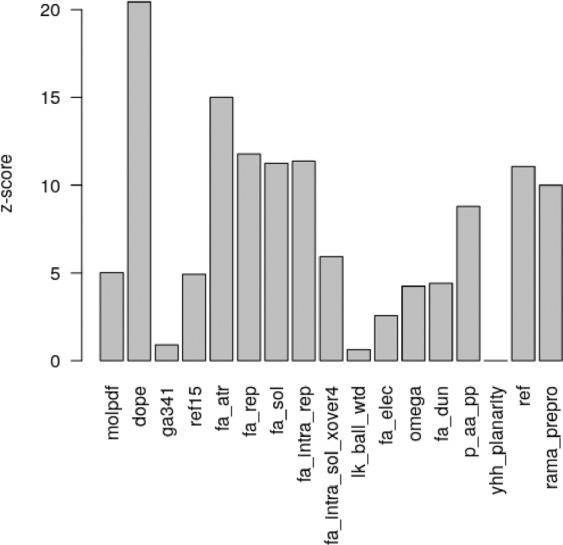
Mean z-scores for energy terms obtained from Boruta feature selection method.

A similar analysis was performed using a random forest classifier and the algorithm Boruta. Figure 5 shows the most important features. DOPE and fa_rep terms showed a large impact on the classification. Since this classifier is more sophisticated and capable of capturing nonlinear relationships in data, more features were identified in this case. The term fa_atr, describing the attractive part of the Lennard-Jones potential between two atoms on different residues separated by a certain distance, approximates van der Waals interactions, which are known to be important for fiber stabilization. A similar interpretation has fa_intra_rep, but it is calculated for atoms within the same residue. Finally, ref is a reference energy for a given amino acid type in an unfolded state and it was introduced in Rosetta as a tool for protein design. It reflects the importance of the amino acid composition of amyloidogenic fragments. Since logistic regression and random forest used slightly different features, we tested if both methods produced consistent results. Comparing the results from both classifiers, it turned out that less than 7% of sequences were classified differently.

## Conclusions

Recognizing amyloidogenic propensity of short peptides provides more knowledge on their potentially adverse behavior, especially if they appear inside longer functional proteins. Bioinformatical methods offering their fast and faultless identification are indispensable tools to advance the prediction of amyloids.

We proposed a new method predicting amyloidogenic propensity of hexapeptides. PATH is based on threading potentially amyloidogenic sequences on zipper-like amyloid structures, corresponding to all representative and experimentally confirmed structural classes of short amyloids. An affinity of a sequence to each structural class was first evaluated with regard to the total energy of its structure. The structures were obtained using comparative modeling with regard to structures of class representatives. A model with the minimal DOPE statistical potential, representing total energy of the structure, was assumed as the most stable and the most accurate for each tested sequence. Although this energy could hint on potential amyloidogenicity of a sequence, since the median energy of amyloids was lower than that of non-amyloids, it did not allow for unambiguous split into two classes. Some of the non-amyloidogenic sequences, forced to assume an amyloid structure, received energies lower than amyloidogenic sequences.

In the other study, only amyloidogenic sequences were considered. The objective was prediction of the most suitable structural class of a query sequence, based on the total energy of its model structure. The correct structural class was accurately predicted for 46% of sequences. The class corresponding to the model with the lowest energy was selected out of 7 possible classes. This analysis, however, could not be regarded as conclusive - the set of available instances is very scarce, containing only 24 sequences. There might be also other reasons which additionally hamper this kind of modeling, such as the choice of the statistical potential, representing energy of the structures, or tendency of amyloid structures to polymorphism.

Since the general structural features are not sufficient for differentiating between amyloids and non-amyloids, in the next stage of the classification we applied more specific and descriptive energy terms from PyRosetta. The extended set of statistical potentials, corresponding to the best model structure from the first stage of modeling, was used to build computational machine learning models. Out of several available algorithms, we finally selected logistic regression, as the one which gave the best accuracy. Additionally, this method is not so much of a black-box type, allowing for interpretation of the results. Our method was verified on two data sets, using also cross-validation. PATH showed a very good potential for classification of amyloidogenicity, with AUC ROC at 0.88, sensitivity *S*_*n*_ = 0.42, specificity *S*_*p*_ = 0.94, and MCC equal to 0.44. The relatively low value of sensitivity is a problem for many other amyloid predictors and is related to the low ratio of amyloids to non-amyloids in the available experimental data. This was the case in our method. Applying the other benchmark data set, consisting of hexapeptides from the pep424 set, the sensitivity was much higher (*S*_*n*_ = 0.8569), without deterioration of the specificity (*S*_*p*_ = 0.9069). This was due to much better balanced number of instances representing both classes in this data set.

Confronting our method with other available predictors, we note that its effectiveness is very high and it could effectively support modeling amyloidogenicity. One of its assets, compared to other methods, is the combination of the structural approach with machine learning on numerous instances. It appears that a somehow similar approach of modeling amyloidogenicity was very recently applied in the version 2.0 of Waltz database class prediction^21^, in which experimental data of the instances are accompanied with their structural models and their energy values. The authors, however, do not reveal all details regarding their method and its performance.

Due to the high interpretability of our method, it was possible to identify the most important features that distinguished amyloids from non-amyloids in the classification. Apart from differences in total energies, such as DOPE from Modeler and REF15 from PyRosetta, some other energy terms appeared to play a role. All applied methods for feature selection showed the importance of the fa_rep energy term, representing repulsive van der Waals interactions, which approximate Pauli repulsion, whose high values may indicate clashes in structures and imply that non-amyloidogenic fragments may not be fitted well into a steric zipper structure. It could explain relatively low accuracy of the first stage of modeling, in which non-amyloids threaded on amyloid structures, are indeed faulty. Also, a relatively high importance of the energy term describing repulsion of atoms within the same residue (fa_intra_rep) was observed. A relatively low importance of statistical terms describing conformation of the backbone and side chains were observed. It should be noted, however, that statistical potentials were mostly fitted to describe structures of globular proteins and their use with other proteins may not be optimal^11^, therefore using better suited descriptors might improve the results and give more insight into the most influential features of the structures.

## Data Availability

All the scripts are available on https://github.com/KubaWojciechowski/PATH.

## References

[1] Eisenberg D, Jucker M (2012). The amyloid state of proteins in human diseases. Cell.

[2] McGlinchey RP, Lee JC (2018). Why study functional amyloids? Lessons from the repeat domain of pmel17. J. molecular biology.

[3] de la Paz ML, Serrano L (2004). Sequence determinants of amyloid fibril formation. Proc. Natl. Acad. Sci..

[4] Eisenberg DS, Sawaya MR (2017). Structural studies of amyloid proteins at the molecular level. Annu. review biochemistry.

[5] Howie AJ, Brewer DB (2009). Optical properties of amyloid stained by congo red: history and mechanisms. Micron.

[6] Nielsen L (2001). Effect of environmental factors on the kinetics of insulin fibril formation: elucidation of the molecular mechanism. Biochemistry.

[7] Shirahama T, Cohen AS (1967). High-resolution electron microscopic analysis of the amyloid fibril. The J. cell biology.

[8] Wang Z (2003). Afm and stm study of b-amyloid aggregation on graphite. Ultramicroscopy.

[9] Sarroukh R, Goormaghtigh E, Ruysschaert J-M, Raussens V (2013). Atr-ftir: a “rejuvenated” tool to investigate amyloid proteins. Biochimica et Biophys. Acta (BBA)-Biomembranes.

[10] Walsh I, Seno F, Tosatto SC, Trovato A (2014). Pasta 2.0: an improved server for protein aggregation prediction. Nucleic acids research.

[11] Ahmed AB, Znassi N, Château M-T, Kajava AV (2015). A structure-based approach to predict predisposition to amyloidosis. Alzheimer’s & Dementia.

[12] Maurer-Stroh S (2010). Exploring the sequence determinants of amyloid structure using position-specific scoring matrices. Nat. methods.

[13] Conchillo-Solé O (2007). Aggrescan: a server for the prediction and evaluation of" hot spots" of aggregation in polypeptides. BMC bioinformatics.

[14] Garbuzynskiy SO, Lobanov MY, Galzitskaya OV (2009). Foldamyloid: a method of prediction of amyloidogenic regions from protein sequence. Bioinformatics.

[15] Gasior P, Kotulska M (2014). Fish amyloid–a new method for finding amyloidogenic segments in proteins based on site specific co-occurence of aminoacids. BMC bioinformatics.

[16] Família C, Dennison SR, Quintas A, Phoenix DA (2015). Prediction of peptide and protein propensity for amyloid formation. PloS one.

[17] Burdukiewicz M (2017). Amyloidogenic motifs revealed by n-gram analysis. Sci. reports.

[18] Emily, M., Talvas, A. & Delamarche, C. Metamyl: a meta-predictor for amyloid proteins. *Plos one* **8** (2013). 10.1371/journal.pone.0079722 PMC3834037 24260292

[19] Tsolis, A. C., Papandreou, N. C., Iconomidou, V. A. & Hamodrakas, S. J. A consensus method for the prediction of ‘aggregation-prone’ peptides in globular proteins. *Plos one* **8** (2013). 10.1371/journal.pone.0054175 PMC3542318 23326595

[20] Wozniak PP, Kotulska M (2015). Amyload: website dedicated to amyloidogenic protein fragments. Bioinformatics.

[21] Louros, N. *et al*. Waltz-db 2.0: an updated database containing structural information of experimentally determined amyloid-forming peptides. *Nucleic Acids Res*.** 48,** D389–D393 (2020). 10.1093/nar/gkz758 PMC6943037 31504823

[22] Reynolds NP (2017). Competition between crystal and fibril formation in molecular mutations of amyloidogenic peptides. Nat. communications.

[23] Beerten J (2015). Waltz-db: a benchmark database of amyloidogenic hexapeptides. Bioinformatics.

[24] Šali A, Blundell TL (1993). Comparative protein modelling by satisfaction of spatial restraints. J. molecular biology.

[25] Alford RF (2017). The rosetta all-atom energy function for macromolecular modeling and design. J. chemical theory computation.

[26] Chaudhury S, Lyskov S, Gray JJ (2010). Pyrosetta: a script-based interface for implementing molecular modeling algorithms using rosetta. Bioinformatics.

[27] Pedregosa F (2011). Scikit-learn: Machine learning in python. J. machine learning research.

[28] Kursa MB, Rudnicki WR (2010). Feature selection with the boruta package. J. Stat Softw.

[29] Tycko R (2015). Amyloid polymorphism: structural basis and neurobiological relevance. Neuron.

[30] Kouza M, Banerji A, Kolinski A, Buhimschi IA, Kloczkowski A (2017). Oligomerization of fvflm peptides and their ability to inhibit beta amyloid peptides aggregation: consideration as a possible model. Phys. Chem. Chem. Phys..

